# A Thermodynamic Framework for Reliability Kinetics

**DOI:** 10.3390/mi17070817

**Published:** 2026-07-07

**Authors:** Joseph B. Bernstein

**Affiliations:** Department of Electrical and Electronic Engineering, Ariel University, Ariel 40700, Israel; josephbe@ariel.ac.il

**Keywords:** reliability physics, degradation kinetics, Gibbs free energy, degradation correlation, power-law degradation, bias temperature instability (BTI), electromigration, lifetime prediction

## Abstract

Empirical power-law relationships are widely used in reliability physics to describe degradation kinetics and predict lifetime. Such behavior appears across diverse failure mechanisms, including time-dependent dielectric breakdown (TDDB), hot-carrier injection (HCI), bias temperature instability (BTI), electromigration (EM), and fatigue. In this work, a thermodynamic framework for reliability kinetics is developed from Gibbs free energy and entropy partitioning, leading to a generalized kinetic equation that incorporates thermal activation, stress acceleration, and accumulated degradation. The formulation introduces two parameters: a stress coefficient, γ, which describes the influence of externally applied stress, and a correlation coefficient, χ, which describes how accumulated degradation influences subsequent degradation. Negative values of χ correspond to self-limiting evolution, positive values correspond to self-amplifying evolution, and χ=0 represents statistically independent accumulation. Representative reliability mechanisms are interpreted within this framework, with TDDB approaching independent evolution, HCI exhibiting weak self-limiting behavior, BTI showing strong self-limiting behavior, and fatigue exhibiting self-amplifying behavior. Electromigration illustrates the complementary role of stress acceleration through γ. The proposed framework provides a common thermodynamic interpretation of empirical power-law degradation kinetics and introduces degradation correlation as a complementary descriptor for reliability modeling and lifetime prediction.

## 1. Introduction

Reliability engineering has traditionally relied on empirical degradation and failure models including the Arrhenius equation, Black’s equation for electromigration, Coffin–Manson fatigue relations, and numerous power-law degradation models. These approaches have proven remarkably successful for lifetime prediction across a wide range of technologies. Nevertheless, the physical origin of the observed kinetic exponents remains an active area of investigation [1,2,3,4].

Most engineers are trained to apply the First Law of Thermodynamics, which governs the conservation and conversion of energy. The design of machines, circuits, and electronic systems is largely concerned with maximizing the conversion of available energy into useful work. Reliability engineering, however, is fundamentally concerned with the Second Law of Thermodynamics. While the First Law describes how systems operate, the Second Law describes how they age. Every real process generates entropy, producing irreversible changes that accumulate over time and ultimately lead to degradation and failure.

The objective of this work is not to propose a specific microscopic failure mechanism. Rather, we seek to develop a thermodynamic interpretation of reliability kinetics based on entropy production and Gibbs free energy. The resulting framework provides a common language for describing self-limiting, independent, and self-amplifying degradation processes observed across microelectronic technologies.

### 1.1. Reliability Engineering and the Second Law

The importance of the Second Law may be illustrated through a simple mechanical example. Consider a rock dropped from height h. The initial potential energy is determined by the gravitational field and the elevation of the object above the ground. Upon impact, the rock comes to rest. No useful mechanical work is recovered. The available energy is dissipated through deformation, sound, heat, and other irreversible processes. In this sense, the impact converts ordered mechanical energy into a larger number of inaccessible microscopic states [5,6,7].

A nearly ideal rubber ball behaves differently. When dropped from the same height, most of the stored energy is recovered as useful mechanical work and the ball rebounds to nearly its original height. Only a small fraction of the energy is dissipated through internal friction, sound, and heating. The entropy generated during each impact is correspondingly small.

An aged rubber ball provides a simple model for degradation. Although the initial potential energy remains unchanged, the rebound height gradually decreases with repeated use. Microstructural changes accumulated during previous impacts increase the irreversible energy loss during each subsequent cycle. The difference between the initial and recovered energy appears as heat, sound, and microscopic structural evolution. Repeated over many cycles, these small irreversible losses accumulate and eventually lead to observable degradation and failure [7].

The Second Law relates such irreversible evolution to entropy. Since the Gibbs free energy depends on the entropy change associated with the degradation process, the present formulation considers only the evolving configurational entropy. In statistical mechanics, this entropy change may be expressed as

(1) $$\Delta S=kln\left(\Omega \right)$$

where Ω represents the multiplicity of accessible microstates associated with the evolving degradation process. In reliability, degradation may therefore be viewed as the progressive evolution of a system toward an increasing number of accessible microscopic configurations governing degradation kinetics [5,8].

### 1.2. Gibbs Free Energy and Reliability

While entropy quantifies the tendency of a system toward disorder, Gibbs free energy determines the energy available to perform useful work. The free energy is given by

(2) ΔG=ΔH−TΔS

where *ΔH* represents the enthalpic contribution and *TΔS* represents the entropic contribution [6,9].

Traditional reliability models often emphasize activation energies associated with specific microscopic mechanisms. However, from a thermodynamic perspective, degradation is governed by the evolution of the free-energy landscape. Entropy production alters the available free energy and therefore influences the kinetics of degradation. If reliability degradation reflects the evolution of a system through an evolving free-energy landscape, the central question becomes how the entropy term changes under applied stress and accumulated damage.

### 1.3. Entropy Partitioning and Reliability Kinetics

Before introducing a mathematical description of the entropy change associated with degradation, it is useful to consider the physical origin of the microscopic states that contribute to degradation. Two conceptually different influences govern the evolution of a degrading system. The first is externally applied driving forces, such as voltage, electric field, current density, temperature, or mechanical stress, which provide the energy required for microscopic evolution. The second is the internal state of the material itself. As degradation accumulates, defects, trapped charge, dislocations, voids, or other structural changes modify the local environment and therefore influence the accessibility of future microscopic states. These external and internal contributions arise from different physical origins and therefore provide a natural basis for separating the thermodynamic description into stress-dependent and degradation-dependent components.

The Second Law of Thermodynamics requires that irreversible processes proceed through an increase in entropy. Since the Gibbs free energy depends on the entropy change associated with degradation, the present formulation considers only the evolving configurational entropy. In reliability degradation, this entropy change reflects the evolution of the accessible microscopic configurations participating in the degradation process. These configurations may correspond to defects, trapped charge, dislocations, voids, bond rearrangements, percolation pathways, or other microscopic structural and electronic states, depending on the degradation mechanism. Although the microscopic details differ among failure mechanisms, the thermodynamic requirement that irreversible evolution proceeds through an evolving set of accessible microscopic configurations is common to all systems as they degrade.

In a degrading material, structure, device, or system, the accessible microstates contributing to the entropy change, Ω, arise from multiple physical contributions, including mechanical, structural, defect, charge, and stress-related influences [2,3,4,10,11]. The total multiplicity may therefore be represented as the product of corresponding sub-ensembles,

(3) $$\Omega =\Omega_{\Psi }\Omega_{Q}$$

where $\Omega_{\Psi }\;$ represents microstates associated with the externally applied stress and $\Omega_{Q}$ represents microstates associated with the accumulated degradation state.

Hence, Equation (3) should be viewed as a first-order thermodynamic approximation rather than an exact statistical identity. Similar separations are routinely employed throughout thermodynamics and statistical mechanics when distinct physical processes contribute independently to the free energy, ΔG. Here, the externally applied stress modifies the thermodynamic driving force for degradation, whereas the accumulated degradation modifies the configurational accessibility of subsequent microscopic states.

Although higher-order coupling between stress and degradation certainly exist in specific materials and devices, this first-order approximation assumes that these contributions remain independent of their corresponding multiplicities and will be treated separately. Such coupling terms may be incorporated in future refinements of the framework but are neglected here to emphasize the dominant physical result reflected in the observed data. So, next we substitute Equation (3) into Equation (1) to get

(4) $$\Delta S=k\;\ln\left(\Omega_{\Psi }\Omega_{Q}\right)=k\;\ln\Omega_{\Psi }+k\;\ln\Omega_{Q}\;,$$

the multiplicative form of Equation (3). This follows directly from the additive nature of entropy changes. Since entropy is an extensive thermodynamic quantity, independent physical contributions are commonly represented by additive entropy terms. The Boltzmann relation, ΔS=kln Ω, therefore implies that multiplicative contributions to the evolving multiplicity become additive contributions to the entropy change. Consequently, the present formulation does not assume multiplicative state counting for the complete microcanonical ensemble. Rather, it only represents the stress-induced, $\Omega_{\Psi }$, and degradation-induced, $\Omega_{Q}$, contributions to the evolving entropy change as separate thermodynamic components.

These functional forms chosen for the stress and degradation multiplicities represent the simplest scale-invariant description capable of reproducing the empirical power-law behavior observed across a broad range of reliability phenomena. Power-law kinetics have been reported empirically for electromigration, fatigue, hot-carrier degradation, bias temperature instability, dielectric breakdown, and numerous other evolving systems. The objective here is therefore not to derive these power laws from microscopic first principles, but rather to identify the simplest thermodynamic representation of the entropy change that is consistent with these widely observed experimental relationships.

A more complete thermodynamic development of the correlation entropy hypothesis, including its historical motivation from Arrhenius, Eyring, Mott, Adler, disorder, occupancy, and evolving free-energy landscapes, together with the derivation of the entropy-change formulation, has recently been presented in [11]. The present perspective summarizes only those elements required for the reliability framework.

The validity of this approximation ultimately depends on the degree of coupling between these processes and should therefore be regarded as a data driven physically motivated modeling assumption. The corresponding stress and degradation sub-ensembles can now be directly represented as

(5) $${\;\Omega_{\Psi }=\left(\frac{\Psi }{\Psi_{0}}\right)}^{\gamma }\;$$

and

(6) $$\Omega_{Q}={\left(\frac{Q}{Q_{0}}\right)}^{\chi }\;$$

where Ψ is the applied stress parameter, Q is the degradation state variable, and γ and χ are scaling exponents. These variables are normalized to initial values Ψ_0_ and Q*_0_* such that at the initial time, t=0, the states will be unity (1) such that the ln(1) = 0. Then, using Equations (4)–(6), the total entropy contribution becomes

(7) $$T\Delta S=\gamma kT\ln\left(\frac{\Psi }{\Psi_{0}}\right)+\chi kT\ln\left(\frac{Q}{Q_{0}}\right)$$

Substituting Equation (7) into Equation (2) and defining $\Delta H=E_{a}$ gives

(8) $$\Delta G=E_{a}-\gamma kT\ln\left(\frac{\Psi }{\Psi_{0}}\right)-\chi kT\ln\left(\frac{Q}{Q_{0}}\right)$$

where $E_{a}$ is the activation energy barrier before including the stress and accumulated-degradation entropy terms. The activation energy $E_{a}$ in Equation (8) represents the intrinsic enthalpic barrier before including entropy contributions. The effective free-energy barrier governing degradation evolves continuously through the stress, and thus, degradation dependent entropy terms. Consequently, while the intrinsic activation energy is treated as constant, the effective barrier ΔG becomes a function of the evolving degradation state Q. This distinction is consistent with the Gibbs free energy formulation developed here.

Following the Eyring formalism [6], the degradation rate dQ/dt is now seen to be proportional to the probability of overcoming the free-energy barrier:

(9) $$\frac{dQ}{dt}=A_{0}\exp\left(-\frac{\Delta G}{kT}\right)$$

where $A_{0}$ contains the remaining proportionality constants. The rate equation therefore becomes

(10) $$\frac{dQ}{dt}=A_{0}exp\left(-\frac{E_{a}}{kT}\right)\;{\left(\frac{\Psi }{\Psi_{0}}\right)}^{\gamma }{\left(\frac{Q}{Q_{0}}\right)}^{\chi }.$$

For constant stress and temperature, Equation (10) is integrated directly for the constant, K, where

(11) $$K=A_{0}exp\;,\left(-\frac{E_{a}}{kT}\right){\left(\frac{\Psi }{\Psi_{0}}\right)}^{\gamma }$$

Then, Equation (10) becomes

(12) $$\frac{dQ}{dt}=KQ^{\chi }\;$$

which is integrated over time to give

(13) $$Q^{\;1-\chi }=Q_{0}^{\;1-\chi }+(1-\chi )Kt\;.$$

This yields the power law evolution in time,

(14) $$Q(t)=Q_{0}\pm {\left[(1-\chi )K\;t\;\right]}^{1/(1-\chi )}$$

where,

(15) $$n=\frac{1}{1-\chi }\;\;,$$

or equivalently,

(16) $$m=\frac{1}{n}=1-\chi \;\;,$$

demonstrating that the experimentally observed power-law exponent follows directly from the thermodynamic correlation coefficient. This derivation provides a direct thermodynamic interpretation of the empirical power-law exponents, m=1/n, widely observed in reliability physics.

The parameter γ seen in Equation (10) describes the influence of applied stress, while χ describes the influence of accumulated degradation. Negative values of χ correspond to self-limiting degradation, positive values correspond to self-amplifying degradation, and χ=0 represents statistically independent accumulation. The examples explored in the following sections demonstrate that all three regimes are observed in practical reliability problems.

## 2. Reliability Models and Lifetime Prediction

Reliability physics has produced numerous microscopic models for degradation mechanisms such as electromigration (EM), time-dependent dielectric breakdown (TDDB), hot-carrier injection (HCI), bias temperature instability (BTI), and fatigue. These models have provided valuable physical insight and often explain specific experimental observations. However, despite decades of investigation, competing physical interpretations frequently coexist for the same degradation mechanism and often result in substantially different lifetime projections.

At the same time, empirical reliability relations have proven remarkably persistent. Examples include Black’s equation for electromigration, Coffin–Manson fatigue relations, and the power-law degradation kinetics widely observed in BTI, HCI and related mechanisms. While the microscopic explanations continue to evolve, these empirical relationships often remain stable across technologies, materials, and generations of devices. This observation suggests that the empirical kinetic behavior itself may contain information that transcends an exclusively energetic barrier interpretation.

The ultimate objective of any reliability model is not merely to describe degradation under accelerated stress, but to predict long-term behavior under operating conditions. Consequently, a useful reliability framework should provide physically meaningful parameters that can be extracted from accelerated measurements and transferred consistently to lifetime projection. Hence, reliability engineers must understand which model parameters control degradation evolution and determine whether those parameters remain valid when extrapolated to operating conditions. The generalized reliability kinetic equation derived in Section 1 suggests that degradation evolution depends on three factors: thermal activation through the activation barrier $E_{a}$, stress acceleration through the coefficient γ, and accumulated degradation through the correlation coefficient χ. Different assumptions regarding degradation correlation may therefore lead to fundamentally different degradation trajectories and lifetime projections.

The coexistence of multiple projection methodologies is not limited to a single degradation mechanism. In TDDB, E-model, 1/E-model, thermochemical, and percolation approaches have all been proposed for lifetime prediction [12,13]. In BTI, reaction–diffusion, trapping, four-state, and non-radiative multiphonon models have been used to explain similar power-law degradation behavior [14,15]. Likewise, HCI degradation is commonly described using empirical power-law relations, although the underlying physical interpretation has been attributed to interface-state generation, charge trapping, and related mechanisms [16,17]. Despite these differences, the observed degradation kinetics often remain remarkably consistent while the physical interpretations continue to evolve without converging to a single universally accepted physical interpretation. As a result, multiple models may adequately describe accelerated test data yet produce substantially different lifetime projections when extrapolated to operating conditions.

Consequently, the central problem is often not whether a model can fit accelerated degradation data, but whether it can accurately predict long-term behavior and MTTF under operating conditions. Equation (10) provides an alternative interpretation. Rather than focusing exclusively on microscopic degradation mechanisms, the generalized kinetic framework indicates that degradation evolution may be governed by three independent quantities:

- Activation energy, $E_{a}$, which determines thermal sensitivity.
- Stress coefficient, γ, which determines stress acceleration.
- Correlation coefficient, χ, which determines how accumulated degradation influences subsequent degradation.

Activation energy and stress acceleration determine how rapidly degradation begins, while the correlation coefficient determines how degradation evolves as damage accumulates. Different assumptions regarding degradation correlation can therefore produce substantially different lifetime projections, even when competing models fit the same accelerated test data.

**Figure 1:**
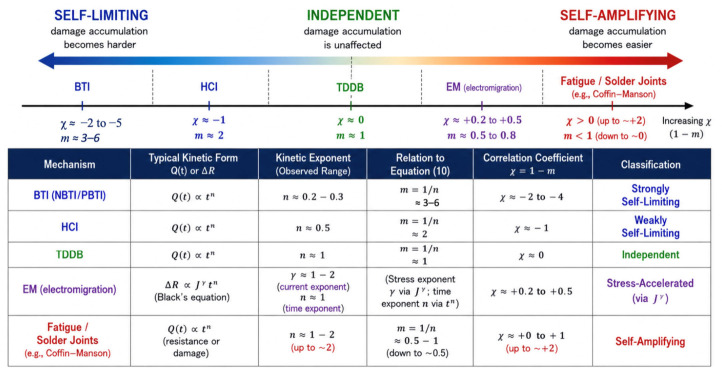
Correlation spectrum classification of common failure mechanisms.

This observation suggests that reliability mechanisms may be classified according to the sign and magnitude of the correlation coefficient χ. Equation (10) naturally gives rise to three distinct classes of degradation evolution: self-limiting evolution (χ < 0), independent evolution (χ = 0), and self-amplifying evolution (χ > 0). These classes are examined in the following section using representative reliability mechanisms and are summarized graphically in Figure 1.

This figure places several common reliability mechanisms along a correlation spectrum ranging from strongly self-limiting evolution (χ < 0) to self-amplifying evolution (χ > 0), with statistically independent accumulation (χ = 0) occupying the intermediate position. Although the precise values of n, m, and χ depend on the specific technology, stress conditions, and degradation metric employed, the relative ordering of these mechanisms provides a useful framework for interpreting reliability kinetics. In the following sections, representative examples from TDDB, HCI, BTI, EM, and fatigue are examined within this correlation-spectrum perspective. Conventional reliability analysis has focused primarily on activation energy and stress acceleration [18,19]. The present framework proposes that degradation correlation represents a third independent descriptor governing how degradation evolves once initiated.

The purpose of this Perspective is not to replace established reliability methodologies, but to provide a common thermodynamic interpretation of the parameters that govern degradation evolution. The detailed thermodynamic development of the correlation framework has recently been presented in [11], while its application to advanced gate-all-around BTI datasets and lifetime projection has been demonstrated separately in [20]. In that work, the activation energy $E_{a}$, stress coefficient γ, and degradation correlation coefficient χ (or equivalently the reciprocal time exponent m=1/n) were extracted directly from accelerated degradation data. The present paper builds upon these complementary developments to provide a unified interpretation applicable across multiple reliability mechanisms.

From an engineering perspective, the proposed framework introduces degradation correlation as an additional descriptor that complements, rather than replaces, conventional reliability parameters. Activation energy determines the thermal sensitivity of degradation, while the stress coefficient describes the influence of the applied operating conditions. The correlation coefficient provides different information: it characterizes how the degradation process itself evolves as damage accumulates. Thus, two degradation mechanisms may exhibit similar activation energies and stress acceleration while following substantially different degradation trajectories because of differences in degradation correlation. Separating these three contributions provides a more complete physical description of degradation evolution and may improve the consistency of long-term lifetime extrapolation.

Conventional reliability studies often focus on when degradation begins and how rapidly it is accelerated by temperature or stress. The present work emphasizes that an equally important question is how degradation evolves in time after it has begun. The correlation coefficient addresses this aspect directly by quantifying whether accumulated degradation promotes, suppresses, or leaves unchanged the probability of subsequent degradation events.

## 3. Correlation Classes of Reliability Evolution

Equation (10) suggests that reliability mechanisms may be classified according to the sign and magnitude of the correlation coefficient χ. In this framework, χ describes how accumulated degradation influences subsequent degradation. Negative values of χ correspond to self-limiting evolution, positive values correspond to self-amplifying evolution, and χ = 0 corresponds to statistically independent accumulation. While activation energy $E_{a}$ and stress coefficient γ determine how rapidly degradation begins, the correlation coefficient χ determines how degradation evolves over time as damage accumulates.

**Figure 2:**
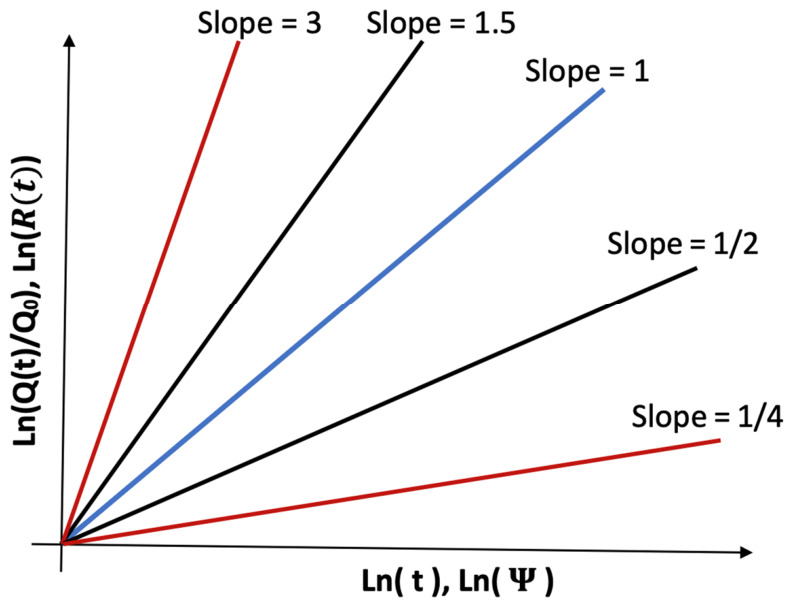
Log-log representation of generalized power-law degradation kinetics. The slope corresponds to either the time exponent n for $Q(t)\propto t^{n}$ or the stress exponent γ for $Q(\Psi )\propto \Psi^{\gamma }$.

The objective of this classification is not to identify the correct microscopic model for a particular failure mechanism. Rather, the goal is to examine whether commonly observed reliability behaviors can be organized according to their degradation kinetics. Different microscopic models may produce similar values of χ, while competing physical interpretations may lead to nearly identical degradation trajectories. Consequently, the correlation coefficient provides a complementary perspective that focuses directly on degradation evolution and lifetime prediction. This perspective suggests that apparently unrelated reliability mechanisms may be organized according to a common degradation-correlation framework. The generalized power-law framework is illustrated in Figure 2, which shows the equivalent log-log representation of time exponents n and stress exponents γ.

The classification presented in Figure 1 is based primarily on the time exponent *n* and the resulting correlation coefficient χ. However, many of these mechanisms also exhibit stress acceleration through a power-law stress exponent γ. Figure 2 illustrates that the generalized kinetic framework contains two complementary power-law contributions: stress acceleration through γ and degradation evolution through χ. The objective here is not to advocate a particular voltage- or stress-acceleration model, but rather to show that a power-law representation of the stress dependence is naturally consistent with the proposed thermodynamic framework.

The correlation coefficient is not intended to identify a unique degradation mechanism. Rather, it provides a complementary kinetic descriptor that describes how accumulated degradation influences subsequent degradation. Reliable mechanism identification generally requires consideration of the activation energy $E_{a}$, stress coefficient γ, the observable degradation parameter, and the underlying physical mechanism. Consequently, similar values of χ may arise for different degradation mechanisms, just as different mechanisms may exhibit similar activation energies or stress exponents. The present framework therefore complements, rather than replaces, conventional reliability analysis by providing an additional thermodynamic perspective on degradation evolution and lifetime prediction.

### 3.1. Independent Evolution: χ ≈ 0

The simplest case corresponds to statistically independent degradation events. In this regime, the probability of a degradation event occurring is independent of previously accumulated degradation. Equation (10) therefore reduces to

(17) $$\frac{dQ}{dt}=A_{0}exp\left(-\frac{E_{a}}{kT}\right){\left(\frac{\Psi }{\Psi_{0}}\right)}^{\gamma }$$

where degradation depends only on the applied stress and activation barrier. The accumulated degradation state Q does not directly influence the subsequent degradation rate.

Time-dependent dielectric breakdown (TDDB) provides a useful example of approximately independent evolution. Numerous microscopic explanations have been proposed, including E-model, 1/E-model, thermochemical, and percolation approaches [12,13]. Despite these differences, many TDDB models describe breakdown as the gradual accumulation of defects until a critical condition is reached.

The percolation model developed by Stathis and co-workers provides a particularly clear illustration of this behavior [18]. In this framework, defects accumulate statistically within the dielectric until a critical defect density produces a conductive path through the oxide. Breakdown occurs when the percolation threshold is reached. The degradation process is therefore governed primarily by defect generation and accumulation rather than by strong feedback from previously accumulated damage.

Independent evolution occupies a special position within the present framework because it corresponds to the conventional assumption employed in many reliability analyses. Activation energy and stress acceleration remain the primary descriptors of degradation, while correlation effects are assumed to be negligible. Consequently, χ ≈ 0 serves as a useful reference point against which self-limiting and self-amplifying degradation mechanisms may be compared.

In TDDB, the degradation variable Q should not be identified with the experimentally observed Weibull distribution of breakdown times. Rather, Q represents the evolving internal degradation state of the dielectric, including the accumulation of defects and the progressive formation of a percolation path. Within the percolation framework, individual defect-generation events are commonly treated as independent occurrences, corresponding, in the present thermodynamic description, to χ≈0. The observed Weibull distribution arises subsequently from the statistical variation in the critical percolation condition among nominally identical devices, rather than from correlated defect generation itself.

The percolation model predicts χ ≈ 0 because defect generation is approximately independent, while the experimentally observed Weibull distribution characterizes the statistical dispersion of the final breakdown condition rather than the correlation of individual degradation events. This fits into our framework as the middle ground where microstate creation is dependent only on external stressors whereas the cumulative effect may be sudden after a critical number of independent defects are created.

### 3.2. Weakly Self-Limiting Evolution: χ < 0

Many reliability mechanisms exhibit degradation kinetics that evolve more slowly than predicted by statistically independent accumulation. In these cases, previously accumulated degradation reduces the rate of subsequent degradation, producing a self-limiting evolution. Within the present framework, this behavior corresponds to a negative value of the correlation coefficient χ.

Hot-carrier injection (HCI) provides a useful example of weakly self-limiting evolution. Experimental studies have long shown that transistor degradation under hot-carrier stress follows a power-law dependence on time,

(18) $$\Delta P=At^{n}$$

where n is typically less than unity. Takeda and Suzuki demonstrated that a wide range of HCI degradation data could be represented by such power-law behavior, establishing one of the earliest empirical reliability models based on degradation kinetics rather than a specific microscopic mechanism [16]. Subsequent work by Sun, Orlowski, and Fu further examined the origin of this behavior and identified parameter correlations consistent with a self-limiting degradation process [17].

Within the generalized kinetic framework of Equation (10), a typical HCI exponent of n≈0.5 corresponds to m≈2 and therefore χ=1−m≈1.

This value places HCI between the independent evolution characteristic of TDDB and the more strongly self-limiting behavior observed in BTI. Physically, the result suggests that accumulated interface damage, trapped charge, or local structural changes progressively reduce the probability of additional degradation events. The degradation process therefore continues, but at a decreasing rate.

The significance of HCI within the present framework is that it represents an intermediate regime between independent and strongly correlated evolution. Unlike TDDB, degradation does not proceed as a purely statistical accumulation of defects. Unlike BTI, however, the correlation remains relatively weak, producing a power-law exponent that remains close to n≈0.5. Consequently, HCI occupies an important transition region within the proposed correlation spectrum and provides a useful illustration of weak negative degradation correlation.

### 3.3. Strongly Self-Limiting Evolution: χ ≪ 0

Bias Temperature Instability (BTI) provides one of the clearest examples of strongly self-limiting degradation evolution. Unlike TDDB, which approaches the independent limit of χ ≈ 0, and HCI, which typically exhibits weak negative correlation, BTI degradation commonly follows power-law kinetics with exponents substantially smaller than unity. Numerous experimental studies have reported threshold voltage shifts that evolve according to

(19) $$\Delta V_{TH}=At^{n}$$

where the time exponent typically falls in the range (n≈0.2−0.3) [14,15,19]. Although the physical interpretation of BTI remains an active area of investigation, the observed power-law behavior has remained remarkably consistent across multiple technologies and experimental conditions.

Within the generalized kinetic framework of Equation (10), these values correspond to

$$m=\frac{1}{n}\approx 3-5$$

which gives

χ=1−m≈−2 to−4.

These values represent the largest negative correlation coefficients among the reliability mechanisms considered in Figure 1. The result suggests that accumulated degradation substantially reduces the probability of subsequent degradation events, producing a strongly self-limiting evolution.

The existence of strongly negative values of χ is particularly interesting because BTI has generated numerous competing physical models over the past two decades. Reaction–diffusion theory, charge trapping models, four-state models, and non-radiative multiphonon descriptions have all been proposed to explain the observed degradation behavior [14,15]. While these models differ significantly in their microscopic assumptions, they frequently reproduce similar power-law degradation trajectories. The persistence of the observed time exponent despite substantial differences in physical interpretation suggests that the degradation kinetics themselves may contain information that is not uniquely tied to any specific microscopic mechanism.

From the perspective of Equation (10), the experimentally observed power-law exponent may be interpreted as a measure of degradation correlation. As charge becomes trapped, defects are generated, or local structural configurations evolve, the remaining accessible microstates become progressively more difficult to occupy. Each additional degradation event therefore contributes less to future degradation than the preceding event. The degradation process continues, but the incremental rate of change gradually decreases. This behavior corresponds naturally to negative values of χ and produces the familiar sublinear power-law evolution observed experimentally.

Recent analyses of advanced gate-all-around (GAA) technologies provide additional support for this interpretation. Multiple independent studies have reported remarkably consistent values of the power-law exponent despite differences in device geometry, measurement methodology, and stress conditions [20]. When expressed within the present framework, these observations correspond to similar values of m and χ across a broad range of experimental conditions. Such consistency suggests that degradation correlation may represent a more fundamental descriptor of BTI evolution than a particular microscopic model.

The present framework does not attempt to replace existing physical descriptions of BTI. Rather, it provides an alternative perspective for interpreting the experimentally observed kinetics. Reaction–diffusion, trapping, four-state, and non-radiative multiphonon models may all remain valuable descriptions of the microscopic processes responsible for degradation. However, the resulting degradation trajectories can also be viewed through the correlation coefficient χ, which quantifies how accumulated degradation influences subsequent degradation. In this sense, χ provides a kinetic descriptor that complements rather than replaces microscopic physical models.

BTI therefore occupies the strongly self-limiting region of the correlation spectrum shown in Figure 1. The large negative values of χ inferred from experimental observations indicate that accumulated degradation substantially suppresses subsequent degradation events. Among the reliability mechanisms considered in this work, BTI provides the clearest example of strong negative degradation correlation and illustrates the importance of considering accumulated damage as an active participant in the degradation process rather than merely a passive consequence of it.

### 3.4. Self-Amplifying Evolution: χ > 0

The opposite extreme of the correlation spectrum occurs when accumulated degradation increases the probability of subsequent degradation events. In this regime, damage acts as a positive feedback mechanism, producing a self-amplifying evolution. Within the present framework, this behavior corresponds to positive values of the correlation coefficient χ.

Mechanical fatigue provides a familiar example of self-amplifying degradation. Repeated stress cycles generate microstructural damage that gradually accumulates within the material. As cracks initiate and propagate, local stress concentrations increase, making additional crack growth progressively easier. The accumulated degradation therefore accelerates subsequent degradation rather than suppressing it. This behavior is commonly described by the Coffin–Manson relation,

(20) $$\begin{array}{cccc} & \frac{\Delta \varepsilon_{p}}{2}=\varepsilon_{f}^{\prime }(2N_{f}{)}^{-c} & & \end{array}$$

where $\Delta \varepsilon_{p}$ is the plastic strain range, $N_{f}$ is the number of cycles to failure, $\varepsilon_{f}^{\prime }$ is the fatigue ductility coefficient, and c is the Coffin–Manson exponent [3,4]. The equation demonstrates that fatigue lifetime follows a power-law dependence on the applied cyclic strain. Similar power-law relations are widely employed in solder-joint reliability, thermal cycling, and other cyclic fatigue phenomena.

The Coffin–Manson relation is an empirical model that has been used extensively to describe fatigue behavior in metals and electronic packaging structures for more than half a century [3,4]. Although the microscopic details differ among materials and structures, the common characteristic is that previously accumulated damage contributes directly to future degradation. Crack initiation, crack propagation, and local stress concentration all increase the likelihood of subsequent damage, producing a naturally self-amplifying degradation process.

Within the generalized kinetic framework of Equation (10), positive values of χ produce degradation trajectories that accelerate with increasing damage. Unlike BTI and HCI, where degradation becomes progressively more difficult as damage accumulates, fatigue mechanisms often exhibit increasing susceptibility to failure as cracks grow and stress concentrations develop. The degradation process therefore evolves toward increasing rates of damage accumulation.

Experimental studies of solder-joint degradation support this interpretation. Measurements of solder-joint resistance during thermal cycling have shown accelerating degradation as crack propagation progresses and conductive pathways are progressively reduced [21]. Similar behavior has been observed in mechanical fatigue, where crack growth rates increase as the effective defect size increases. These observations are consistent with positive degradation correlation and place fatigue mechanisms within the self-amplifying region of the correlation spectrum.

Fatigue therefore provides a useful counterexample to the self-limiting behavior observed in BTI and HCI. While self-limiting mechanisms exhibit negative values of χ and progressively slower degradation, fatigue exhibits positive values of χ and progressively faster degradation. Together, these mechanisms illustrate the broad range of degradation trajectories that emerge naturally from the generalized kinetic framework.

The continued use of multiple accepted projection methodologies demonstrates the difficulty of identifying a unique physical description from accelerated test data alone. Reliability standards such as JEP-122 recognize this challenge by presenting multiple accepted approaches for several common failure mechanisms [22]. The generalized thermodynamic framework presented here suggests that these apparently different models may instead reflect different combinations of the activation energy, stress coefficient, and correlation coefficient that govern degradation kinetics.

### 3.5. Stress Acceleration and the ‘γ’ Exponent

The preceding examples illustrate the role of the correlation coefficient χ in determining how degradation evolves with accumulated damage. Equation (10), however, contains a second power-law term associated with the applied stress through the stress exponent γ. Reliability engineers routinely employ stress-acceleration relations in which degradation rate depends on current density, voltage, electric field, mechanical strain, or other externally applied stresses.

Electromigration provides one of the most familiar examples through Black’s equation [2],

(21) $$MTTF=AJ^{-n}exp\left(\frac{E_{a}}{kT}\right)$$

where the current-density exponent n describes the sensitivity of lifetime to applied stress. Current-density exponents between approximately 1 and 2 are commonly employed in practical electromigration lifetime prediction and remain a central component of modern reliability qualification methodologies [2,22]. More recent reviews continue to identify current density as one of the primary determinants of electromigration reliability and treat Black’s equation as a foundational electromigration model [23].

Unlike the correlation coefficient χ, which describes how accumulated degradation influences subsequent degradation, the stress exponent γ describes how external conditions modify the degradation rate. Within the present framework, the stress term

$${\left(\frac{\Psi }{\Psi_{0}}\right)}^{\gamma }$$

arises naturally from the stress-related entropy contribution represented by $\Omega_{\Psi }$. The resulting acceleration law has the same mathematical form as many widely used empirical reliability relations, including current-density acceleration in electromigration, voltage acceleration in TDDB and BTI, and stress acceleration in fatigue and fracture processes.

The importance of stress acceleration is illustrated by the continuing discussion surrounding the appropriate value of the electromigration current exponent. Although Black originally proposed n=2, subsequent investigations have reported a range of values depending on temperature, stress conditions, interconnect geometry, and the dominant transport mechanisms [24]. Despite these differences, the power-law dependence itself remains remarkably robust and continues to serve as a practical basis for lifetime prediction. This situation closely parallels the discussion of degradation correlation presented in the preceding sections. The numerical values of the exponents may vary, while the underlying power-law form remains broadly applicable.

Electromigration also provides an example of the interaction between stress acceleration and degradation evolution. Classical electromigration models describe atomic transport driven by current density and temperature, while later studies recognized the coupled interaction between mass transport, stress gradients, resistance changes, and Joule heating [25]. These coupled effects introduce feedback mechanisms that influence degradation evolution even though the dominant acceleration factor remains the applied current density.

Electromigration occupies a unique position within the present framework because it is commonly characterized by both a stress exponent and a degradation trajectory. Black’s equation emphasizes the current-density acceleration term, whereas later studies recognized that stress evolution, void growth, and resistance increase can introduce additional feedback mechanisms during degradation. Consequently, electromigration demonstrates that stress acceleration and degradation correlation are not mutually exclusive concepts. A reliability mechanism may exhibit strong stress sensitivity through γ while simultaneously developing degradation correlation through χ as damage accumulates.

This generalized kinetic framework therefore contains two distinct power-law contributions. The stress exponent γ describes how external conditions modify the degradation rate, while the correlation coefficient χ describes how accumulated degradation modifies subsequent degradation. Both terms influence lifetime prediction, but they represent different physical processes. Stress acceleration determines how rapidly degradation begins, whereas degradation correlation determines how degradation evolves after it begins.

This distinction explains why reliability mechanisms with similar activation energies may exhibit substantially different degradation trajectories. It also explains why mechanisms with similar degradation kinetics may display very different stress sensitivities. Equation (10) incorporates both effects within a common thermodynamic framework and provides a unified description of stress acceleration and degradation evolution.

## 4. Implications for Reliability Modeling and Lifetime Prediction

The examples presented in Section 3 suggest that a wide range of reliability mechanisms may be organized within a common degradation-correlation framework. Although TDDB, HCI, BTI, EM, and fatigue are typically treated as distinct physical phenomena, they exhibit degradation trajectories that can be interpreted using the same generalized kinetic equation. Figure 1 illustrates this relationship by placing these mechanisms along a correlation spectrum ranging from strongly self-limiting evolution to self-amplifying evolution.

Traditionally, reliability models have focused on identifying the microscopic processes responsible for degradation. This approach produces a physical insight and leads to the development of numerous successful predictive models. At the same time, competing physical interpretations often coexist for the same degradation mechanism. TDDB, HCI, and BTI all provide examples where multiple accepted physical descriptions can explain similar experimental observations while leading to vastly different assumptions regarding long-term degradation behavior and MTTF extrapolations.

The correlation spectrum shown in Figure 1 provides a useful perspective for interpreting reliability kinetics. Mechanisms such as TDDB occupy the nearly independent limit where degradation evolves primarily through statistical accumulation. HCI exhibits weak negative correlation, while BTI represents a strongly self-limiting process in which accumulated degradation progressively reduces the probability of subsequent degradation events. At the opposite extreme, fatigue and crack-growth phenomena exhibit positive correlation, where accumulated damage accelerates future degradation. Electromigration occupies a unique position because it is commonly characterized by both stress acceleration and degradation evolution, demonstrating that the stress coefficient γ and the correlation coefficient χ represent distinct but complementary aspects of reliability behavior.

The primary objective of reliability modeling is lifetime prediction. In practice, engineers are often less concerned with identifying a unique microscopic mechanism than with accurately predicting time-to-failure under operating conditions. Different physical models may reproduce accelerated degradation data with comparable accuracy while producing substantially different lifetime projections when extrapolated beyond the measurement range. The present framework suggests that degradation correlation may represent an important factor in this divergence. Models that imply different values of χ will produce different degradation trajectories even when they describe the same accelerated data set.

The proposed framework does not replace existing reliability models. Rather, it provides a complementary perspective for interpreting degradation kinetics and comparing apparently unrelated failure mechanisms. By introducing degradation correlation as a common descriptor, Equation (10) provides a unified thermodynamic interpretation of empirical power-law behavior across a broad range of reliability phenomena. This perspective may help explain why similar degradation trajectories appear repeatedly across different technologies, materials, and failure mechanisms, while simultaneously emphasizing the central importance of degradation evolution in lifetime prediction.

## 5. Conclusions

This work developed a thermodynamic framework for reliability kinetics based on Gibbs free energy and entropy partitioning. Beginning with the Boltzmann relation for entropy and the Gibbs free-energy formalism, a generalized kinetic equation was derived that incorporates thermal activation, stress acceleration, and accumulated degradation within a common mathematical framework. The resulting expression results in the common empirical power-law relationships widely observed in reliability physics.

The formulation introduces two parameters that characterize degradation evolution. The stress coefficient, γ, describes the influence of externally applied stress, while the correlation coefficient, χ, describes how accumulated degradation influences subsequent degradation. Negative values of χ correspond to self-limiting evolution, positive values correspond to self-amplifying evolution, and χ=0 corresponds to statistically independent accumulation.

Several representative reliability mechanisms were examined within this framework. TDDB was shown to approximate independent evolution, HCI to exhibit weak self-limiting behavior, and BTI to represent strongly self-limiting degradation. In contrast, fatigue and crack-growth phenomena were shown to exhibit self-amplifying behavior. Electromigration provided an example in which both stress acceleration and degradation evolution contribute to reliability extrapolation. Together, these examples demonstrate that apparently unrelated physical mechanisms may be organized within a common degradation-correlation spectrum.

The proposed framework does not replace existing microscopic reliability models. Rather, it provides a complementary perspective for interpreting degradation kinetics and comparing different reliability mechanisms using a common set of thermodynamic descriptors. By emphasizing degradation correlation as a fundamental characteristic of reliability evolution, the framework helps explain the widespread appearance of empirical power-law behavior across diverse materials, technologies, and failure mechanisms.

Because the primary objective of reliability physics is accurate lifetime prediction, the correlation coefficient may provide a useful additional descriptor alongside activation energy and stress acceleration. The generalized kinetic equation developed here suggests that degradation evolution itself can play a central role in determining long-term reliability behavior and may provide a common thermodynamic language for comparing reliability mechanisms and improving lifetime extrapolation across diverse technologies.

## Data Availability

No new data was presented in this study.

## References

[1] Arrhenius S. (1889). Über die Reaktionsgeschwindigkeit bei der Inversion von Rohrzucker durch Säuren. Z. Phys. Chem..

[2] Black J.R. (1969). Electromigration—A Brief Survey and Some Recent Results. IEEE Trans. Electron Devices.

[3] Coffin L.F. (1954). A Study of the Effects of Cyclic Thermal Stresses on a Ductile Metal. Trans. ASME.

[4] Manson S.S. (1953). Behavior of Materials Under Conditions of Thermal Stress. NASA Report 1170. http://hdl.handle.net/2060/19930083626.

[5] Kittel C., Kroemer H. (1980). Thermal Physics.

[6] Eyring H. (1935). The Activated Complex in Chemical Reactions. J. Chem. Phys..

[7] Bernstein J.B., Bensoussan A., Bender E. (2024). Reliability Prediction for Microelectronics.

[8] Boltzmann L. (1877). Über die Beziehung zwischen dem zweiten Hauptsatze der mechanischen Wärmetheorie und der Wahrscheinlichkeitsrechnung respektive den Sätzen über das Wärmegleichgewicht. Sitzungsberichte Kais. Akad. Wiss..

[9] Gibbs J.W. (1873). A Method of Geometrical Representation of the Thermodynamic Properties of Substances by Means of Surfaces. Trans. Conn. Acad. Arts Sci..

[10] Bernstein J.B. (2025). Power-Law Reliability Plotting for Microelectronics. Micromachines.

[11] Bernstein J.B. (2026). Correlation Entropy and Power-Law Kinetics. Entropy.

[12] Lombardo S., Crupi F., Campisano G., Neri B., Nicollian E.H., Irrera F. (2005). Dielectric Breakdown Mechanisms in Gate Oxides. J. Appl. Phys..

[13] Stathis J.H. (2001). Percolation Models for Gate Oxide Breakdown. Microelectron. Reliab..

[14] Schroder D.K., Babcock J.A. (2003). Negative Bias Temperature Instability: Road to Cross in Deep Submicron Silicon Semiconductor Manufacturing. J. Appl. Phys..

[15] Grasser T., Kaczer B., Goes W., Reisinger H., Aichinger T., Hehenberger P., Wagner P.-J., Schanovsky F., Franco J., Roussel P. (2011). The Paradigm Shift in Understanding the Bias Temperature Instability: From Reaction–Diffusion to Switching Oxide Traps. IEEE Trans. Electron Devices.

[16] Takeda E., Suzuki N. (1983). An Empirical Model for Device Degradation Due to Hot-Carrier Injection. IEEE Electron Device Lett..

[17] Sun S.W., Orlowski M., Fu K.-Y. (1990). Parameter Correlation and Modeling of the Power-Law Relationship in MOSFET Hot-Carrier Degradation. IEEE Electron Device Lett..

[18] Stathis J.H., DiMaria D.J. (1999). Reliability Projection for Ultra-Thin Oxides at Low Voltage. IEDM Technical Digest.

[19] Palumbo F., Lombardo S., Crupi F. (2022). Reliability and Physics of Degradation in Advanced CMOS Technologies. Microelectron. Reliab..

[20] Bernstein J.B. (2026). Lifetime Projection for BTI Assuming Non-Constant Power Law Exponent. Microelectron. Reliab..

[21] Gershman I., Bernstein J.B. (2013). Nondestructive Quantitative Analysis of Crack Propagation in Solder Joints. IEEE Trans. Compon. Packag. Manuf. Technol..

[22] JEDEC Solid State Technology Association (2011). JEP122G: Failure Mechanisms and Models for Semiconductor Devices.

[23] Cheng P., Mao L.-F., Shen W.-H., Yan Y.-L. (2025). Electromigration Failures in Integrated Circuits: A Review of Physics-Based Models and Analytical Methods. Electronics.

[24] Huang X., Kteyan A., Tan S.X.-D., Sukharev V. (2016). Physics-Based Electromigration Models and Full-Chip Assessment for Power Grid Networks. IEEE Trans. Comput.-Aided Des. Integr. Circuits Syst..

[25] Young D., Christou A. (1994). Failure Mechanism Models for Electromigration. IEEE Trans. Reliab..

